# Supplementary figures and images for: MicroRNA-705 regulates the differentiation of mouse mandible bone marrow mesenchymal stem cells
Source: PeerJ. 2019 Jan 10;7:e6279. doi: 10.7717/peerj.6279 (PMC6330203; doi:10.7717/peerj.6279)

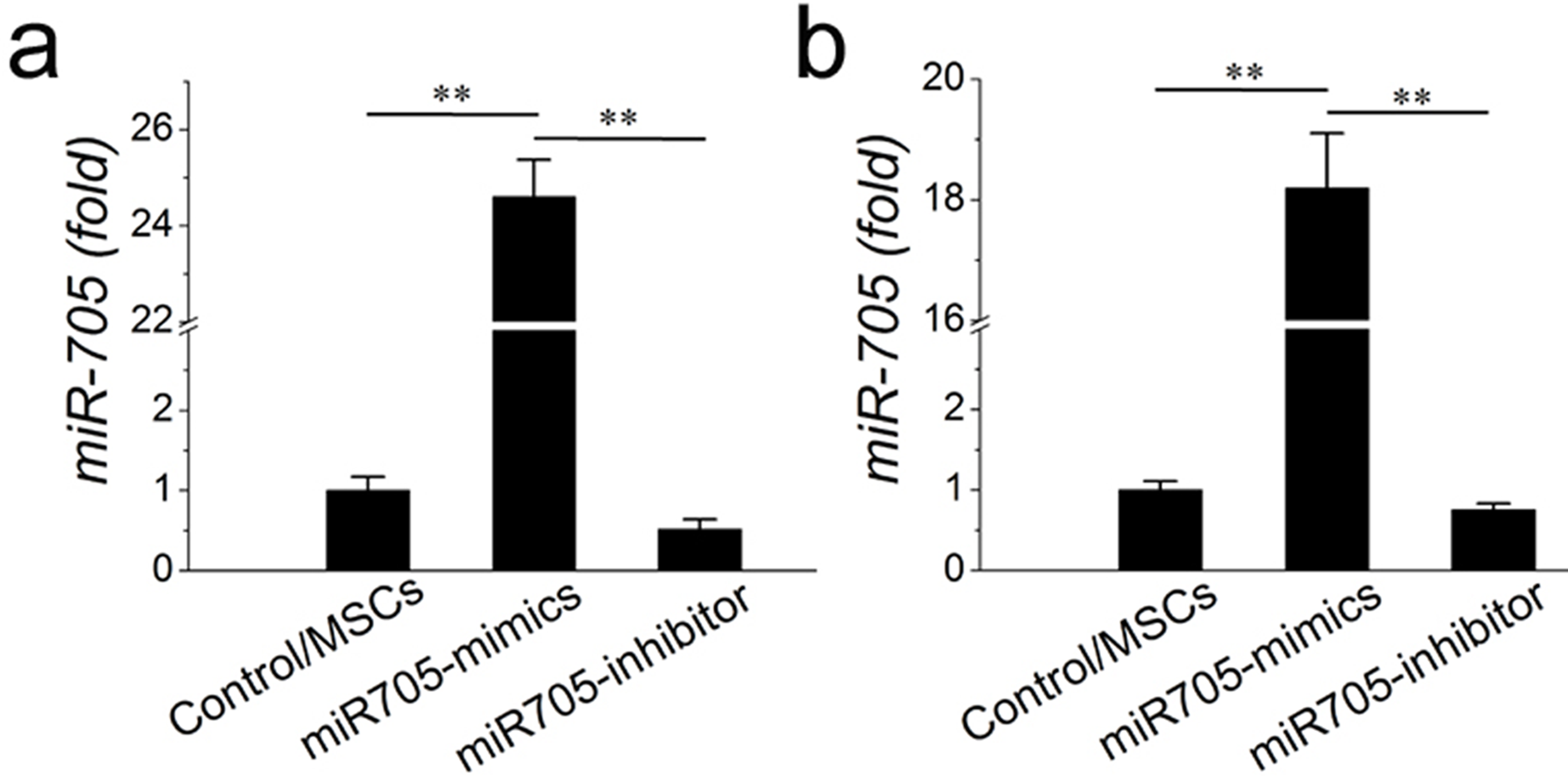

Supplement: Figure S1 — q RT-PCR was used to confirm the effectiveness of miR705 inhibitor at 14-day of osteogenic induction (A) and at 7-day of adipogenic induction (B). Values are described as mean ± SD from three independent experiments. * P < 0.01, ** P < 0.001. [file peerj-07-6279-s001.png]
