# Supplementary material for: MicroRNA-705 regulates the differentiation of mouse mandible bone marrow mesenchymal stem cells
Source: PeerJ. 2019 Jan 10;7:e6279. doi: 10.7717/peerj.6279 (PMC6330203; doi:10.7717/peerj.6279)
Supplement: Data S1 — Table 1 shows the sequence of five pairs of primer used in the q RT-PCR. [file peerj-07-6279-s002.docx]

**Supplementary table 1**

| **Gene** | **Sequence (5'>3')** |
| --- | --- |
| *ppar-γ* | **Forward primer** CCGAGTCTGTGGGGATAAAGC  **Reverse primer** GGTCTCTCGGAAACTCCCTTTG |
| *lpl* | **Forward primer** AGCCCTTGCTAGGAGAAAGC  **Reverse primer** ATAATGGGGATGCCGGTGAC |
| *runx2* | **Forward primer** CCTCACAAACAACCACAGAACC  **Reverse primer** CAAAACAAAACGGAGTGAGCAAATA |
| *alp* | **Forward primer** CTTCATAAGCAGGCGGGGGA  **Reverse primer** GAGCCCAGATGGTGGGAAGA |
| *β-actin* | **Forward primer** CTGGCACCACACCTTCTACA |
|  | **Reverse primer** GGTACGACCAGAGGCATACA |
